# Relationship of residency program characteristics with pass rate of the American Board of Internal Medicine certifying exam

**DOI:** 10.3402/meo.v20.28631

**Published:** 2015-09-29

**Authors:** Amporn Atsawarungruangkit

**Affiliations:** Department of Family Medicine, Rajavithi Hospital, Bangkok, Thailand

**Keywords:** ABIM, pass rate, program characteristics, internal medicine residency

## Abstract

**Objectives:**

To evaluate the relationship between the pass rate of the American Board of Internal Medicine (ABIM) certifying exam and the characteristics of residency programs.

**Methods:**

The study used a retrospective, cross-sectional design with publicly available data from the ABIM and the Fellowship and Residency Electronic Interactive Database. All categorical residency programs with reported pass rates were included. Using univariate and multivariate, linear regression analyses, I analyzed how 69 factors (e.g., location, general information, number of faculty and trainees, work schedule, educational environment) are related to the pass rate.

**Results:**

Of 371 programs, only one region had a significantly different pass rate from the other regions; however, as no other characteristics were reported in this region, I excluded program location from further analysis. In the multivariate analysis, pass rate was significantly associated with four program characteristics: ratio of full-time equivalent paid faculty to positions, percentage of osteopathic doctors, formal mentoring program, and on-site child care (OCC). Numerous factors were not associated at all, including minimum exam scores, salary, vacation days, and average hours per week.

**Conclusions:**

As shown through the ratio of full-time equivalent paid faculty to positions and whether there was a formal mentoring program, a highly supervised training experience was strongly associated with the pass rate. In contrast, percentage of osteopathic doctors was inversely related to the pass rate. Programs with OCC significantly outperformed programs without OCC. This study suggested that enhancing supervision of training programs and offering parental support may help attract and produce competitive residents.

In 2015, 26,252 residents were admitted to the first year of a residency program. The largest type of residency program – categorical internal medicine – has 6,698 positions (1). Medical students and physicians are required to pass multiple examinations (e.g., United States Medical Licensing Examination [USMLE], Comprehensive Osteopathic Medical Licensing Examination of the United States [COMLEX-USA]) to get into a residency program. Moreover, physicians must pass a board exam to be eligible to practice in their medical specialty. Likewise, to practice as an internist, a physician must pass the American Board of Internal Medicine (ABIM) certifying exam, which had an average pass rate for first takers of 86% from 2012 to 2014 (2). Failing this exam doubtlessly has an impact on a physician's career plan, as the exam is only given once a year.

Passing the ABIM exam is important for physicians regardless of their gender. In the past 30 years, the gender distribution of the global physician community has changed considerably; specifically, in both the United States and global contexts, the percentage of female physicians has been continuously increasing (3, 4). Residency programs must therefore promptly adapt to this changed scenario. On the residents’ side, numerous factors can relate to the selection process of residency programs, such as the location of the program, program type, educational tracks, or compensation. Some medical graduates might have specific preferences in this regard, such as a women's health track, on-site child care (OCC), or subsidized child care. However, as no studies have directly assessed how these program characteristics differently associate with the pass rate, it would be important to clarify this point.

Previous studies on what variables relate to pass rate have looked primarily at the relationship between pass rate and exam scores (i.e., for in-training examinations or USMLE) (5–11) or the effect of duty hour reform (12–14). Unfortunately, neither factor is directly related to the characteristics of a residency program, because the former is an important predictor of individual performance whereas the latter is a regulatory factor. Some evidence suggests that location and program size are associated with the pass rate of the American Board of Family Medicine (ABFM) (15) and American Board of Pediatrics (ABP) certifying exams (16). Moreover, recent studies have shown that the pass rate of the ABFM certifying exam was also associated with accreditation cycle length, opportunities for international experiences, and training in alternative medicine (17).

Besides location and program size, there is limited evidence on the relationships between pass rate and the characteristics of internal medicine residency programs. Thus, the objective of the present study is to evaluate the relationships between the pass rate of the ABIM certifying exam and the characteristics of three-year categorical internal medicine residency programs. Because the educational environment plays a crucial role in the success of medical education (18) and creating competitive residents (19), understanding these relationships will help to improve the quality of residency education and should be beneficial for various stakeholders, including program directors, residents, residency candidates, and patients.

## Methods

This study used a retrospective, cross-sectional design to evaluate the relationships between pass rate of the ABIM certifying exam and most of the program characteristics available in the Fellowship and Residency Electronic Interactive Database (FREIDA^®^), a freely available online database containing self-reported program characteristics. The scope of this study covered all three-year categorical internal medicine residency programs in the United States and Puerto Rico, a US territory. A list of three-year categorical internal medicine programs and their characteristics were extracted from FREIDA^®^ using computerized automation on April 24, 2015. The 2012–2014 pass rates of the ABIM certifying exam, the most recent statistics at the time of the study, were obtained from the ABIM website. Residency programs that did not report their pass rates were excluded from the study.

The internal medicine residency programs were classified into 10 regions as listed in the FREIDA^®^ (Table 1). Program size was defined as the average number of residency positions from postgraduate year 1 to 3. I used only the USMLE score requirements for interviews in this study as most residency candidates had taken this exam rather than the COMLEX-USA. Salary and vacation days were taken from first-year data only because this year had the greatest number of data observations. Hard-to-quantify data (e.g., sick days, call schedules, and average USMLE Step 1 score) were not taken into account in this analysis. The number of program faculty members was excluded from the analysis because the ratio of full-time equivalent paid faculty to positions (FTP ratio) provides more meaningful information in this regard. Finally, the visa qualifications of international medical graduates and major medical benefits were excluded, as they did not seem much relevant to the pass rate. In total, 69 program characteristics in a variety of categories were considered in this study, such as location, general information, number of faculty and trainees, work schedule, educational environment, education benefits, education features, program evaluation, resident evaluation, employment policies and benefits, and compensation and leave.

**Table 1:** Regional locations of internal medicine programs

		Number of programs with pass rate	
		
Regional location	State	All	≥1 characteristics other than location
Mid Atlantic	New Jersey, New York, Pennsylvania	95	73
East North Central	Illinois, Indiana, Michigan, Ohio, Wisconsin	67	55
South Atlantic	Delaware, District of Columbia, Florida, Georgia, Maryland, North Carolina, South Carolina, Virginia, West Virginia	53	42
Pacific	California, Hawaii, Oregon, Washington	38	25
New England	Connecticut, Maine, Massachusetts, New Hampshire, Rhode Island, Vermont	35	33
West South Central	Arkansas, Louisiana, Oklahoma, Texas	28	25
West North Central	Iowa, Kansas, Minnesota, Missouri Nebraska, North Dakota, South Dakota	19	19
East South Central	Alabama, Kentucky, Mississippi, Tennessee	15	13
Mountain	Arizona, Colorado, Idaho, Montana, Nevada, New Mexico, Utah	13	10
Territory	Puerto Rico	8	0
Total		405	371

First, the descriptive statistics of the program characteristics were calculated. Then, to identify the relationships between pass rate and the program characteristics, univariate, linear regression analyses were performed under the assumption that all variables were normally distributed. Program characteristics with a *p*-value of less than 0.10 in the univariate analysis were included in the multivariate, linear regression analyses. Then, stepwise, multivariate, linear regression analysis was performed to identify the significant independent predictors of pass rate. The significance level (α) was 0.05. All statistical analyses were conducted using STATA version 13.0 (StataCorp).

## Results

There were a total of 405 three-year categorical internal residency programs in FREIDA^®^ at the time of study; however, only 371 programs (*n*=371) were included in the analysis because the other programs did not report a pass rate. As shown in Table 2, the pass rate of one region, a US territory containing only the state of Puerto Rico, was significantly lower than those of other regions (*p*<0.001). However, because the programs in this region did not report any other program characteristics in FREIDA^®^, I could not analyze any relationships between other characteristics and the pass rate. As such, the factor of location was dropped from further univariate and multivariate analyses.

**Table 2:** ABIM pass rate and regional location of residency program

Regional location	Number of programs	Mean±SD	*p*
Mid Atlantic	95	87.05±8.20	0.107
East North Central	67	84.61±10.73	0.325
South Atlantic	53	85.83±7.95	0.884
Pacific	38	85.81±9.04	0.914
New England	35	86.63±10.02	0.523
West South Central	28	85.44±8.03	0.905
West North Central	19	87.13±8.20	0.487
East South Central	15	83.16±9.81	0.302
Mountain	13	88.51±6.72	0.271
Territory	8	69.22±21.39	<0.001
All locations	371	85.65±9.51	

ABIM=American Board of Internal Medicine.

Among the 371 programs that reported a pass rate, 295 programs (79.51%) reported at least one program characteristic other than location. The baseline characteristics of these programs are summarized in Table 3. Based on the univariate, linear regression analysis of 70 program characteristics (Table 4), 7 characteristics showed a statistically significant association with pass rate: program size (*β*=0.1348, *p*<0.001), university-based program (*β*=2.2413, *p*=0.040), offering preliminary positions (*β*=−2.2413, *p*=0.048), FTP ratio (*β*=0.8977, *p*=0.045), percentage of doctors of osteopathic medicine (% DO; *β*=−0.1356, *p*=0.010), formal mentoring program (FMP; *β*=5.0446, *p*=0.021), and OCC (*β*=3.2413, *p*=0.003).

**Table 3:** Baseline characteristics of residency programs

Program characteristics	Obs.	Mean±SD or number (%)
Pass rate of ABIM certifying exam	295	85.81±9.02
General information		
Program size	295	21.03±12.44
Program type		
University-based	295	106 (35.93)
Community based university affiliated hospital	295	156 (52.88)
Community-based	295	32 (10.85)
Military-based	295	1 (0.34)
Offers preliminary positions	295	202 (68.47)
Minimum score of USMLE Step 1 for interview consideration	243	206.3±11.27
Minimum score of USMLE Step 2 for interview consideration	181	209.01±10.97
Faculty and trainee information		
Full-time paid female physician faculty (%)	292	32.98±13.87
Ratio of full-time equivalent paid faculty to positions	295	1.36±1.17
US medical graduate (%)	229	40.69±34.04
International medical graduate (%)	229	49.68±34.98
Doctor of osteopathic (%)	229	9.38±11.28
Female (%)	229	43.9±8.57
Work schedule information		
Average hours/week on duty^a^,^b^	295	61.97±6.35
Maximum consecutive hours on duty^a^,^b^	295	16.37±3.33
Average number of 24-h off duty periods per week^b^	295	1.28±0.27
Program allows moonlighting^c^	295	213 (72.2)
Night float system (in or beyond first year)	295	279 (94.58)
Offers awareness and management of fatigue in residents	295	295 (100)
Educational environment		
Average hours/week of regularly scheduled lectures/conferences^b^	295	8.01±2.36
Training at hospital outpatient clinics^b^	281	0.24±0.11
Training in ambulatory non-hospital community-based settings^b^	220	0.11±0.09
Educational benefits		
Physician impairment prevention curriculum	295	266 (90.17)
Integrative medicine curriculum	295	55 (18.64)
Debt management/financial counseling	295	226 (76.61)
Formal program to develop teaching skills	295	283 (95.93)
Formal mentoring program	295	277 (93.9)
Formal program to foster interdisciplinary teamwork	295	225 (76.27)
Continuous quality improvement training	295	294 (99.66)
International experience	295	152 (51.53)
Resident retreats	295	240 (81.36)
Off-campus electives	295	267 (90.51)
Hospice/home care experience	295	270 (91.53)
Cultural competence awareness	295	287 (97.29)
Instruction in medical Spanish or other non-English language	295	69 (23.39)
Alternative/complementary medicine curriculum	295	139 (47.12)
Economics of health-care systems curriculum	295	202 (68.47)
MPH/MBA or PhD training	295	50 (16.95)
Required research rotation	274	47 (17.15)
Educational features		
Offers additional training beyond accredited length	295	26 (8.81)
Offers a primary care track	295	113 (38.31)
Offers a rural track	295	2 (0.68)
Offers a women's health track	295	13 (4.41)
Offers a hospitalist track	295	49 (16.61)
Offers a research track/non-accredited fellowship	295	49 (16.61)
Offers another track	295	55 (18.64)
Resident evaluation		
Yearly specialty in-service examination required	295	295 (100)
Patient surveys	295	283 (95.93)
Portfolio system	295	246 (83.39)
360 degree evaluations	295	294 (99.66)
Objective structured clinical examinations (OSCE)	295	199 (67.46)
Program evaluation		
Program graduation rates	295	284 (96.27)
Resident assessment of curriculum	295	255 (86.44)
In-training examination scores	295	294 (99.66)
Performance-based assessment scores	295	245 (83.05)
Employment policies and benefits		
Part-time/shared positions	295	17 (5.76)
On-site child care	295	101 (34.24)
Subsidized child care	295	27 (9.15)
Allowance/stipend for professional expenses	295	285 (96.61)
Leave for educational meetings/conferences	295	246 (83.39)
Moving allowance	295	47 (15.93)
Housing stipend	295	22 (7.46)
On-call meal allowance	295	284 (96.27)
Free parking	295	220 (74.58)
PDAs	295	103 (34.92)
Placement assistance upon completion of program	295	176 (59.66)
Cross coverage in case of illness/disability	295	294 (99.66)
Policy prohibits hiring smokers/users of nicotine products	295	32 (10.85)
Compensation and leave		
Salary compensation^b^ (USD)	285	51909.81±3914.27
Vacation days^b^	295	18.13±3.99

a Excluding beeper call

b during first year

c beyond first year

Obs.=observation.

**Table 4:** Results of the univariate linear regression analysis between the pass rate and program characteristics

Program characteristics	Coefficient (standard error)	*p*
General information		
Program size	0.1348 (0.0416)	0.001*
Program type		
University-based	2.2413 (1.0889)	0.040*
Community based university affiliated hospital	−1.7193 (1.0494)	0.102
Community-based	−0.3514 (1.6920)	0.836
Military-based	−15.8615 (9.0058)	0.079
Offers preliminary positions	−2.2305 (1.1250)	0.048*
Minimum score of USMLE Step 1 for interview consideration	0.0080 (0.0521)	0.879
Minimum score of USMLE Step 2 for interview consideration	0.0287 (0.0651)	0.660
Faculty and trainee information		
Full-time paid female physician faculty (%)	−0.0069 (0.0380)	0.855
Ratio of full-time equivalent paid faculty to positions	0.8977 (0.4466)	0.045*
U.S. medical graduate (%)	0.0333 (0.0173)	0.056
International medical graduate (%)	−0.0166 (0.0170)	0.329
Doctor of osteopathic (%)	−0.1356 (0.0519)	0.010*
Female (%)	0.1045 (0.0691)	0.132
Work schedule information		
Average hours/week on duty^a^,^b^	0.1127 (0.0827)	0.174
Maximum consecutive hours on duty^a^,^b^	0.0492 (0.1581)	0.756
Average number of 24-h off duty periods per week^b^	−0.2992 (1.9767)	0.880
Program allows moonlighting^c^	0.2288 (1.1745)	0.846
Night float system (in or beyond first year)	−3.9024 (2.3122)	0.093
Offers awareness and management of fatigue in residents	0 (N/A)	N/A
Educational environment		
Average hours/week of regularly scheduled lectures/conferences^b^	0.2111 (0.2229)	0.344
Training at hospital outpatient clinics^b^	1.2489 (4.9136)	0.800
Training in ambulatory non-hospital community-based settings^b^	−1.9703 (6.6846)	0.768
Educational benefits		
Physician impairment prevention curriculum	−0.6246 (1.7670)	0.724
Integrative medicine curriculum	0.1754 (1.3511)	0.897
Debt management/financial counseling	−0.1174 (1.2431)	0.925
Formal program to develop teaching skills	2.6572 (2.6592)	0.318
Formal mentoring program	5.0446 (2.1785)	0.021*
Formal program to foster interdisciplinary teamwork	1.4558 (1.2340)	0.239
Continuous quality improvement training	−10.6570 (9.0319)	0.239
International experience	1.7695 (1.0478)	0.092
Resident retreats	−1.1893 (1.3493)	0.379
Off-campus electives	0.3391 (1.7952)	0.850
Hospice/home care experience	2.3139 (1.8846)	0.221
Cultural competence awareness	2.0633 (3.2374)	0.524
Instruction in medical Spanish or other non-English language	2.1183 (1.2369)	0.088
Alternative/complementary medicine curriculum	1.0675 (1.0523)	0.311
Economics of health-care system curriculum	1.6189 (1.1286)	0.153
MPH/MBA or PhD training	1.6615 (1.3992)	0.236
Required research rotation	1.7339 (1.4468)	0.232
Educational features		
Offers additional training beyond accredited length	2.6965 (1.8495)	0.146
Offers a primary care track	1.0512 (1.0807)	0.331
Offers a rural track	−2.5422 (6.4108)	0.692
Offers a women's health track	0.0893 (2.5638)	0.972
Offers a hospitalist track	−2.6849 (1.4052)	0.057
Offers a research track/non-accredited fellowship	2.5240 (1.4062)	0.074
Offers another track	1.2318 (1.3492)	0.362
Resident evaluation		
Yearly specialty in-service examination required	0 (N/A)	N/A
Patient surveys	0.8295 (2.6633)	0.756
Portfolio system	0.0578 (1.4139)	0.967
360 degree evaluations	−8.8168 (9.0386)	0.330
Objective structured clinical examinations (OSCE)	−1.4211 (1.1200)	0.206
Program evaluation		
Program graduation rates	3.5523 (2.7696)	0.201
Resident assessment of curriculum	−1.4542 (1.5347)	0.344
In-training examination scores		
Performance-based assessment scores	−1.2818 (1.4005)	0.361
Employment policies and benefits		
Part-time/shared positions	4.0813 (2.2454)	0.070
On-site child care	3.2413 (1.0927)	0.003*
Subsidized child care	1.2915 (1.8233)	0.479
Allowance/stipend for professional expenses	−1.2586 (1.6905)	0.457
Leave for educational meetings/conferences	−0.7218 (1.4133)	0.610
Moving allowance	−2.0531 (1.4328)	0.153
Housing stipend	2.8371 (1.9962)	0.156
On-call meal allowance	4.3438 (2.7657)	0.117
Free parking	−0.8020 (1.2076)	0.507
PDAs	−1.7615 (1.0990)	0.110
Placement assistance upon completion of program	0.2196 (1.0726)	0.838
Cross coverage in case of illness/disability	−5.1188 (9.0484)	0.572
Policy prohibits hiring smokers/users of nicotine products	−1.2586 (1.6905)	0.457
Compensation and leave		
Salary compensation^b^ (USD)	0.0002 (0.0001)	0.139
Vacation days^b^	−0.1012 (0.1321)	0.444

a Excluding beeper call

b during first year

c beyond first year; N/A, not applicable.

* *p*<0.05.

In the multivariate, linear regression model (Table 5), only four program characteristics were significantly related to pass rate: FTP ratio (*β*=1.2541, *p*=0.015), % DO (*β*=−0.1468, *p*=0.004), FMP (*β*=5.6318, *p*=0.018), and OCC (*β*=2.8760, *p*=0.018). The adjusted *R*^2^ of this multivariate model was 9.61%.

**Table 5:** Multivariate linear regression of the ABIM pass rate and significantly associated program characteristics

Program characteristics	Coefficient (standard error)	*p*
Ratio of full-time equivalent paid faculty to positions	1.2541 (0.5131)	0.015
Doctor of osteopathic (%)	−0.1468 (0.0501)	0.004
Formal mentoring program	5.6318 (2.3543)	0.018
On-site child care	2.8760 (1.2079)	0.018
Constant	78.9831 (2.3967)	<0.001

ABIM=American Board of Internal Medicine; adjusted *R*^2^ of the model is 0.0961.

## Discussions

The study findings are distinct from those of similar studies focusing mostly on location and program size. Although the locations of residency programs have been reported to be significantly related with the pass rate of board certifying exams (ABFM and ABP) (15, 16), the only region in this study found to have a significant relation with the pass rate was Puerto Rico. However, none of the residency programs in this region reported any other program characteristics. As a result, the location factor was excluded from further analysis. Therefore, to my limited knowledge, this is the first time that location has not been reported as a significant predictor of the ABIM pass rate.

Many investigators have previously demonstrated that program size is a significant predictor of the pass rate on board certifying exams of many specialties, including internal medicine (15, 16, 20–22). In the univariate analysis, I confirmed that program size was also a significant predictor (*p*<0.001). However, in the multivariate analysis, FTP ratio was considered a better predictor of pass rate, which conforms to the results of one previous study (23). Compared to program size alone, the FTP ratio contains information from a number of faculty positions relative to program size, which is very similar to student–faculty ratio. It is important to understand that student–faculty ratio is a popular measure of educational quality in higher education (24) and is used by global ranking agencies such as QS Quacquarelli Symonds (25) and US News Ranking (26). In other words, a small residency program might not be a disadvantage as long as the program has a sufficient FTP ratio, which is a better indicator of educational quality.

Of the 295 programs that reported information on FMP, 277 programs (93.9%) offered it. Thus, even if a mentoring program in internal medicine is unstructured, under-monitored, or under-evaluated (27), it appears to have a significant positive relation with pass rate. Some previous researchers looked at the effectiveness of mentoring programs, commonly concluding that both residents and program directors had positive attitudes toward mentoring programs (27–31). However, there are no previous investigations on the relationship between FMP and pass rate of board certifying exams. As such, this seems an interesting area of future exploration.

Interestingly, the % DO was found to be the only significant negative predictor of the pass rate in the multivariate, linear regression. My findings are perhaps explainable by the fact that higher % DO in a given program is an indicator of lower competition in that program. As shown in the case of general surgery residency programs, more competitive programs are significantly more likely to select applicants with higher USMLE Step 1 scores (32), which are also a significant predictor of passing the ABIM certifying exam (8). Thus, the % DO can be seen as an inverse indicator of competitiveness. In this study, I also noted that the percentage of US medical graduates was positively associated with pass rate; however, it was not statistically significant. To my limited knowledge, there have been no empirical comparisons in academic performance between doctors of osteopathic medicine and doctors of medicine in the past.

Only 34.24% of internal medicine residency programs offered an OCC benefit, despite the fact that parenthood during residency is common together with the rising number of residents having babies during residency training (33–35). Having an OCC or another parental support policy could be a critical factor for attracting competitive residents to a program. Indeed, one study on general surgery programs across Canada suggested that a lack of program-specific maternity/parenting policies could lead to lower interest among candidates (36). Moreover, I found that programs with OCC had slightly lower % DO, which would further benefit the pass rate. Such evidence indicates that programs with OCC are more competitive because they can attract more US medical graduates.

Several limitations of this study need to be addressed. First, the data from both the FREIDA^®^ and the ABIM website could be subject to human error by the data reporters or data gatherers. Second, approximately one-fourth of programs opted out of reporting program characteristics other than location to FREIDA. Although such reporting behavior could lead to further bias in the data, the difference in pass rate between fully opted-in programs and fully opted-out programs in this study was found to be non-significant. Third, the study used data from the FREIDA^®^ at a single point of time, and thus the program characteristics and their relations with pass rate might differ by time period. Fourth, the adjusted *R*^2^ of 9.61% means that 90.39% of the variance in pass rates is attributable to factors other than FTP ratio, % DO, FMP, and OCC. Further analysis with a larger dataset would be useful to validate the study results, especially regarding the influence of location using multivariate analysis. Despite the limitations of the data and low adjusted *R*^2^, the findings of this study can still be applied to most programs in the United States (except those located in Puerto Rico). Therefore, the results of this study have several implications for improving internal residency programs. First, programs should focus on improving the supervision of training experiences such as enhancing the quality of the mentoring program or balancing the faculty to position ratio. Second, programs should pay more attention to improving parental support during residency, such as implementing OCC or other facilities for supporting parenthood during residency training.

## Conclusions

According to the results, success on board certifying exams is associated with two main factors: the competitiveness of individual residents and the training environment. Doubtlessly these factors are related. Specifically, the significant findings about FTP ratio and FMP supported the benefits of a well-supervised training environment, whereas higher % DO had a negative effect on pass rate. Finally, the OCC was directly related to training environment in terms of quality of life. The result of this study suggested that internal medicine residency programs could better attract competitive residents into programs as well as produce competitive residents by enhancing the supervision of training environments and offering parental support.
